# The discovery of a selective and potent A_2a_ agonist with extended lung retention

**DOI:** 10.1002/prp2.134

**Published:** 2015-05-04

**Authors:** Annika B M Åstrand, Eva Lamm Bergström, Hui Zhang, Lena Börjesson, Therese Söderdahl, Cecilia Wingren, Anne-Helene Jansson, Amir Smailagic, Camilla Johansson, Håkan Bladh, Igor Shamovsky, Anders Tunek, Tomas Drmota

**Affiliations:** 1 RIA iMed, AstraZeneca R&D MölndalSE-431 59, Mölndal, Sweden; 2 Drug Safety & Metabolism, AstraZeneca R&D MölndalSE-431 59, Mölndal, Sweden; 3 AstraZeneca R&D LundSE-221 87, Lund, Sweden

**Keywords:** A_2a_, Adenosine receptor, blood pressure, heart rate, inflammation, lung retention, pharmacokinetic-pharmacodynamic, therapeutic window

## Abstract

Although the anti-inflammatory role of the A_2a_ receptor is well established, controversy remains with regard to the therapeutic value for A_2a_ agonists in treatment of inflammatory lung diseases, also as a result of unwanted A_2a_-mediated cardiovascular effects. In this paper, we describe the discovery and characterization of a new, potent and selective A_2a_ agonist (compound 2) with prolonged lung retention and limited systemic exposure following local administration. To support the lead optimization chemistry program with compound selection and profiling, multiple in vitro and in vivo assays were used, characterizing compound properties, pharmacodynamics (PD), and drug concentrations. Particularly, pharmacokinetic-PD modeling was applied to quantify the effects on the cardiovascular system, and an investigative toxicology study in rats was performed to explore potential myocardial toxicities. Compound 2, in comparison to a reference A_2a_ agonist, UK-432,097, demonstrated higher solubility, lower lipophilicity, lower plasma protein binding, high rat lung retention (28% remaining after 24 h), and was efficacious in a lung inflammatory rat model following intratracheal dosing. Despite these properties, compound 2 did not provide a sufficient therapeutic index, that is, separation of local anti-inflammatory efficacy in the lung from systemic side effects in the cardiovascular system. The plasma concentration that resulted in induction of hypotension (half maximal effective concentration; EC_50_ 0.5 nmol/L) correlated to the in vitro A_2a_ potency (rIC_50_ 0.6 nmol/L). Histopathological lesions in the heart were observed at a dose level which is threefold above the efficacious dose level in the inflammatory rat lung model. In conclusion, compound 2 is a highly potent and selective A_2a_ agonist with significant lung retention after intratracheal administration. Despite its local anti-inflammatory efficacy in rat lung, small margins to the cardiovascular effects suggested limited therapeutic value of this compound for treatment of inflammatory lung disease by the inhaled route.

## Introduction

Extracellular adenosine exerts a protective role in conditions of stress, cellular damage and injury via increase of tissue perfusion and associated anti-inflammatory effects. These physiological outcomes are the result of activation of four known adenosine receptor subtypes; A_1_, A_2a_, A_2b_, and A_3,_ with all of them being seven transmembrane spanning G-protein coupled receptors (Gessi et al. 2011). The A_2a_ receptor is expressed in various immune cells (i.e., neutrophils, macrophages, T cells, and natural killer cells) and its activation modulates cell trafficking, cell activity, viability and release of inflammatory mediators (Harada et al. 2000; Hasko et al. 2000; Chen et al. 2013). Moreover, a role for A_2a_ in the attenuation of inflammation and tissue damage has also been demonstrated in A_2a_ knockout mice (Ohta and Sitkovsky 2001).

**Figure 1:**
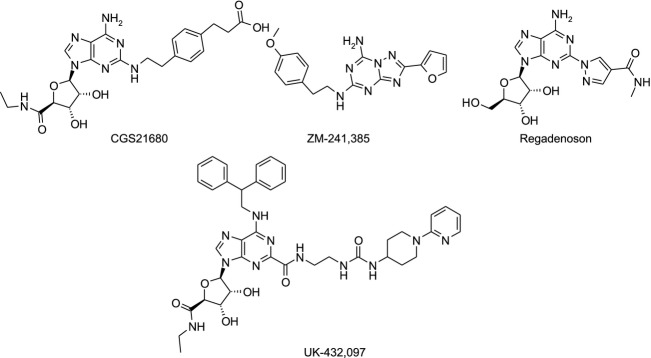
Published A_2a_ agonists and antagonist. CGS21680 (agonist): 3-[4-[2-[[6-amino-9-[(2R,3R,4S,5S)-5-(ethylcarbamoyl)-3,4-dihydroxy-tetrahydrofuran-2-yl]purin-2-yl]amino]ethyl]phenyl]propanoic acid. Regadenoson (agonist): 1-[6-amino-9-[(2R,3R,4S,5R)-3,4-dihydroxy-5-(hydroxymethyl)tetrahydrofuran-2-yl]purin-2-yl]-*N*-methyl-pyrazole-4-carboxamide. UK-432,097 (agonist): 6-(2,2-diphenylethylamino)-9-[(2R,3R,4S,5S)-5-(ethylcarba-moyl)-3,4-dihydroxy-tetrahydrofuran-2-yl]-*N*-[2-[[1-(2-pyridyl)-4-piperidyl]carbamoylamino]ethyl]purine-2-carboxamide. ZM-241,385 (antagonist): 2-(2-furyl)-N5-[2-(4-methoxyphenyl)ethyl]-[1,2,4]triazolo[1,5-a][1,3,5]triazine-5,7-diamine.

Adenosine also induces vasodilatation, especially in the coronary vessels (Stepp et al. 1996). This effect is due to binding of adenosine to the A_2a_ receptor, and this mechanism is supported by studies with selective A_2a_ agonists (Webb et al. 1990, 1991; Mathôt et al. 1995; Nekooeian and Tabrizchi 1996). Lexiscan® (Regadenoson, Astellas Pharma US, Inc, Northbrook, IL, Fig.1) is currently the only selective A_2a_ agonist on the market, where its rapid and short-lasting effect is used during radionuclide myocardial perfusion imaging to determine coronary fractional flow reserve (Johnson and Peters 2010; Al Jaroudi and Iskandrian 2012).

Furthermore, it has also been highlighted that A_2a_ agonists are protective in several ischemia-reperfusion organ injury studies at concentrations well below those required to produce a hypotensive effect (Linden 2001; Fredholm et al. 2002).

Because of its dual physiological function, selectively targeting A_2a_ as a chronic anti-inflammatory treatment without concomitant cardiovascular effects has been extremely challenging. It has been speculated that A_2a_ stimulation by localized drug administration in the pulmonary environment would be beneficial for inflammatory lung conditions, such as asthma and chronic obstructive pulmonary disease (COPD), while reducing the risk of systemically induced cardiovascular side effects (Caruso et al. 2013). Since both effects are governed by the A_2a_ receptor, one plausible way forward was to increase lung retention of the drug while minimizing systemic exposure after inhalation.

In this paper, we describe the properties of compound 2 (Fig.3), a highly potent (in vitro EC_50_ 1.4 nmol/L) and selective (>1000-fold over A_1_, A_2b_ and A_3_) A_2a_ agonist with proven in vitro/in vivo anti-inflammatory efficacy and high retention in the rat lung after intratracheal (i.t.) instillation (28% of dose remaining in lung after 24 h and a terminal half-life in lung of 53 h). Compound 2 has eminent properties compared to the known clinical candidates (UK-432,097) and tool compound (CGS21680). These favorable properties of compound 2 did not help to separate the anti-inflammatory effect in the lung from systemic vasodilatation. Our thorough work suggested there is limited therapeutic value for inhaled A_2a_ agonists in the treatment of inflammatory lung diseases.

## Materials and Methods

### Synthesis of compounds

Compounds 1–3 were synthesized according to Scheme14 The pyridinium compounds were isolated as trifluoro acetic acid salts after high-performance liquid chromatography (HPLC) purification.

**Figure 14:**
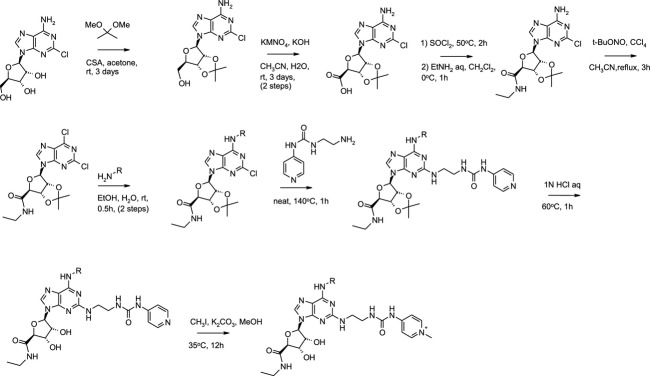
Scheme Synthetic scheme for preparation of compounds 1–3.

### Molecular modeling

Putative binding mode of compound 2 in the agonistic conformation of the A_2a_ receptor was attained by molecular modeling. The structure of the human A_2a_ receptor and the initial binding mode of compound 2 were formed from the X-ray structure of the complex of the receptor with UK-432,097 (Xu et al. 2011; Brokhaven Protein Data Bank, identification code 3QAK). The molecular mechanical energy minimization of the complex of compound 2 with the receptor was performed in the set of geometric variables using the OPLS-2005 force-field (Kaminski et al. 2001) and the implicit Generalized Born water salvation model (Still et al. 1990) as incorporated in the MacroModel program (Maestro 9.5.014; Schrödinger, LLC, New York, NY, 2013). Orientation and conformation of compound 2 and conformations of the side chains of the receptor residues located within 6 Å from the ligand comprised the set of geometric variables.

### In vitro assays

#### Overview of the assays used in the present study

1. *Human A*_*2a*_ *and A*_*2b*_ *receptor pharmacology:* Chinese hamster ovary CHO-K1 cells stably expressing human A_2a_ and A_2b_ receptors were used to determine substances agonistic potency and intrinsic activity. The generation of cAMP in serial dilutions of compound was measured by an Alpha screen assay (cAMP Alphascreen assay kit; Perkin-Elmer, Waltham, MA) and the signal of 10 *μ*mol/L NECA (N-ethyladenosine-5′-uronic acid) represented the maximal cAMP response (i.e., 100% of intrinsic activity) produced by these cells.
2. *Human A*_*1*_ *receptor pharmacology:* A membrane preparation expressing the adenosine A_1_ receptor (Perkin-Elmer) was used to determine the substances agonistic potency and intrinsic activity in the GTP*γ* ^35^[S] filter assay-same layout as the GTP-text below (point 3). The signal corresponding to adenosine at 100 *μ*mol/L was used to define 100% intrinsic activity.
3. *Human A*_*3*_ *receptor pharmacology:* A_3_ receptor G_i_ coupling was measured by GTP*γ* ^35^[S] filter assay at serial dilutions of compound using membranes from Perkin-Elmer. The signal corresponding to the maximal response of NECA at 100 *μ*mol/L was set to 100% intrinsic activity.
4. Lipopolysaccharide (LPS)*-induced TNFα production in primary human peripheral blood mononuclear cells (hTNFα PBMC) and rat mononuclears (rTNFα splenocytes):* Human PBMC and rat mononuclear spleen cells were incubated with serial dilutions of compound and stimulated with LPS. TNF*α* release was measured by ELISA (Enzyme-Linked Immunosorbent Assay).
5. *Plasma protein binding:* The rat plasma protein binding was determined at a compound concentration of 10 *μ*mol/L by equilibrium dialysis against a phosphate buffer and analyzed using liquid chromatography-tandem mass spectrometry.
6. *Lipophilicity:* The octanol–water partition coefficient (LogD) was measured using the shake flask method, phases separated by centrifugation and analyzed by HPLC.
7. *Substance solubility:* Solubility was measured in phosphate buffer at pH 7.4 using dry compounds in solid state.

All details about the different in vitro studies are in Data S1.

### In vivo methods

All animal studies were approved by the local ethical committee in Lund (M311/09) or in Gothenburg (10/2011, M31/11, 267/2011). Male (Pharmacokinetic, PK, studies) or female (Pharmacodynamic, PD, and toxicology studies) Wistar Hannover GALAS rats (Taconic, Silkeborg, Denmark, 226–250 g at arrival) were kept in a facility with 12 h light dark cycle at 21 ± 2°C and with 55 ± 15% relative humidity. R70 diet (Lantmännen, Stockholm, Sweden) and tap water were supplied ad libitum during all studies.

#### Overview of the study and experimental designs

1. Standard in vivo PK studies were performed after oral (p.o.), intravenous (i.v.) and intratracheal (i.t.) dosing where serial blood samples were taken from a surgically implanted catheter in the *arteria carotis* (Popovic procedure) or as a terminal sample from the jugular vein up to 24 h after dosing.
2. In the duration of effect study, the compound or vehicle was given prophylactically by i.t. instillation to 69 rats; 48, 36, 30, 24, and 12 h prior to the LPS challenge and inflammatory cells in the broncheoalveolar lavage (BAL) fluid were investigated 24 h after LPS instillation.
3. In the dose–response study, 98 naïve rats were given the compound or vehicle i.t. 24 h prior to the LPS challenge and inflammatory cells in the BAL fluid were investigated 24 h after LPS instillation.
4. In the cardiovascular investigative study, eight conscious rats in a swivel system were given the compound or vehicle as a four-step i.v. infusion in order to investigate the PK–PD relationship with regard to hypotension (mean arterial blood pressure) and tachycardia (heart rate, HR).
5. Two studies were conducted with repeated dosing in order to investigate the risk of tachyphylaxia; a telemetry study where 24 rats were given the compound or vehicle i.t. for five consecutive days and sacrificed 4 h after the last dose to investigate the hypotensive responses; and an efficacy study where the compound or vehicle was administered i.t. for one, three, or five consecutive days to 120 naïve rats followed by an LPS challenge 24 h after the last dose to investigate the recruitment of inflammatory cells in BAL fluid.
6. In addition, we performed an investigative toxicology study in 19 naïve rats given either a single i.t. dose or repeated dosing at two dose levels of compound 2 for four consecutive days.
7. Moreover, naïve satellite animals were used to capture time points for PK evaluation not available from the PD animals above.

The compounds in all studies were administered as solutions in the specified vehicles given together with all details about the different in vivo studies in the Data S1.

### Data analysis

#### PK–PD analysis of blood pressure and HR responses

The relationship between plasma concentrations of compound 2 and mean arterial pressure (MAP) and HR, respectively, were modeled using data generated in the swivel study, including data from start of first infusion up to 2 h after end of last infusion (Dose 1–4, equivalent to 1.9, 11, 67 and 410 *μ*g/kg per min for 15 min at each step). The baseline value, calculated as average of measurements during the 60 min baseline recording period was used as the predose value. Temporal differences between effect measurements and blood sampling for plasma analysis were accounted for by fitting a standard two-compartment PK model to the time-concentration profile for each individual animal, and subsequently using the individual parameter estimates to simulate compound 2 plasma concentrations at the times of effect measurements. Phoenix® WinNonlin® Version 6.2.1 (Certara, L.P., Princetown, NJ) was used for modelling.

#### Evaluation of efficacy

The efficacy of the compound on neutrophils in BAL fluid is expressed as per cent inhibition of the full window (negative veh/veh controls vs. the positive veh/LPS controls): Inhibition (%) of LPS-induced BAL neutrophils by compound 2 was calculated using the following formula:

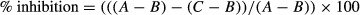
where *A* is the average in the group administered with vehicle and challenged with LPS; *B* is the average in the group administered with vehicle and challenged with saline; and *C* is the average in the group administered with test compound and challenged with LPS.

Blood pressure and HR effects were compared to each rat’s individual baseline pressure (mmHg) and HR (bpm) expressed as a % of baseline.

#### Statistical evaluations

All data are averaged and presented as mean ± SD, unless given as individual values with means. Evaluation of differences of statistical significance was performed by means of one-way analysis of variance followed by Dunnett’s multiple comparison test versus control (GraphPad Prism v.4, Groningen, Netherlands). A *P*-value of <0.05 was considered of statistical significance.

The BAL neutrophil count was expressed on a log scale to better fit a normal distribution. A linear model was fitted (SAS v.9.2, Cary, NC) with the treatment group as the factor of interest, and adjusted by inclusion of a study occasion factor. The interaction between study occasion and treatment group was checked not to have any significant effect and thereafter excluded. Group comparisons, each dose group against the vehicle, were performed as described above.

A mixed effect model with fixed effects for time after dose, treatment groups, and number of doses, was set up for the analysis of the blood pressure and HR data (SAS v.9.2). A random intercept was included to allow for individual baseline blood pressures, as well as a random effect on the number of doses.

## Results

### The development of a new inhaled A_2a_ agonist

**Figure 2:**
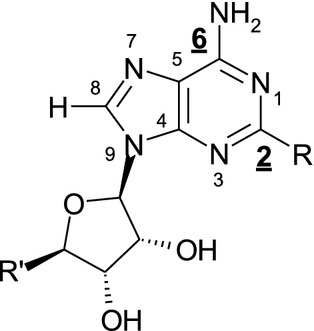
Numbering of A_2a_ agonists. Positions C2 and N6 (highlighted) were subjected to the optimization.

**Figure 3:**
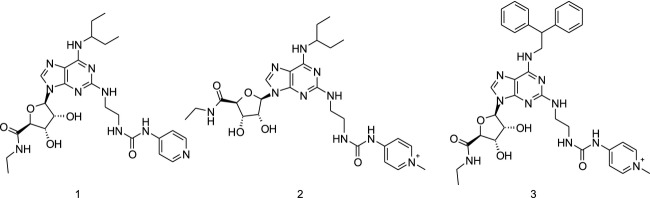
AstraZeneca new compounds. 1: (2S,3S,4R,5R)-*N*-ethyl-5-[6-(1-ethylpropylamino)-2-[2-(4-pyridylcarbamoylamino)ethylamino]purin-9-yl]-3,4-dihydroxy-tetrahydrofuran-2-carboxamide. 2: (2S,3S,4R,5R)-*N*-ethyl-5-[6-(1-ethylpropylamino)-2-[2-[(1-methylpyridin-1-ium-4-yl)carbamoylamino]ethylamino]purin-9-yl]-3,4-dihydroxy-tetrahydrofuran-2-carboxamide. 3: (2S,3S,4R,5R)-5-[6-(2,2-diphenylethylamino)-2-[2-[(1-methylpyridin-1-ium-4-yl)carbamoylamino]ethylamino]purin-9-yl]-*N*-ethyl-3,4-dihydroxy-tetrahydrofuran-2-carboxamide.

The medicinal chemistry synthetic optimization focused on the position C2 and N6 (Fig.2) with the aim to discover a compound with high A_2a_ potency, high selectivity, low lipophilicity (logD), and high solubility. The 1-ethylpropyl substituents on N6 (compound 1 and 2, Fig.3) gave good A_2a_ selectivity over A_1_, A_2b,_ and A_3_ and were found to be optimal for potency whilst keeping the logD low and solubility high (Tables3). Introduction of a positive charge in compound 2 via methylation of the pyridine in the position 2 of compound 1 increased rat lung *t*_½_ to 53 h, and additionally such modification did not compromise the other required properties (Fig.3; Tables3). Compound 2 in comparison with UK-432,097 has low logD (less than −1), high solubility (>1000 *μ*mol/L), low plasma protein binding, and rat lung *t*_½_ increased by 13-fold (Table1). The in vitro human A_2a_ potency was 1.4 nmol/L and selectivity over the other human adenosine receptors was >1000-fold for compound 2 (Tables2 and 3). Furthermore, compound 2 showed similar potency in two biological effect assays that is, inhibition of LPS stimulated TNF*α* release in human PBMC (EC_50_ = 0.5 nmol/L) and rat splenocytes (EC_50_ = 0.6 nmol/L) confirming its anti-inflammatory effect via A_2a_ activation in a native cellular system (Tables2 and 3). Selectivity of compound 2 was tested across 98 targets in binding assays (www.ricerca.com). The only identified significant hits (i.e., >50% effect at 10 *μ*mol/L of compound 2) were A_2a_ (∼100%) and A_3_ (∼84%) data on AstraZeneca file.

**Table 1:** Physicochemical and in vivo pharmacokinetic properties

Compound	LogD	Solubility (*μ*mol/L)	f_u_ rat (%)	Rat blood clearance (mL/min per kg)	Rat oral F (%)	Lung *t*_½_ (h)	Lung/blood split (fold)
CGS21680	< -1	ND	17	26	ND	1	<0.08
UK-432,097	3.4	12	1.4	37	ND	4	2.7
1	2	>1000	20	65	1.4	6	1.8
2	< -1	>1000	44	19	0.3	53	460
3	−0.35	420	11	42	ND	80	1800

**Figure 4:**
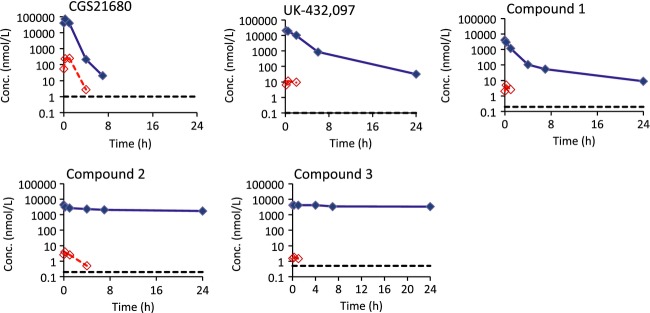
Systemic spillover and lung duration after intratracheal (i.t.) instillation differs between different A_2a_ agonists. Lung concentrations (blue filled diamonds and solid lines) and blood concentrations (red unfilled diamonds and dashed lines) following intratracheal administration of CGS21680, UK-432,097 and compounds 1, 2 and 3 to rats. The black dotted lines show the lower limit of quantification, which was 0.1–1 nmol/L. The dose was 300 *μ*g/kg for CGS21680, 130 *μ*g/kg for UK-432,097 and 17, 17 and 20 *μ*mol/kg for Compounds 1, 2, and 3, respectively. Data are means of *n* = 2/time point.

Values for blood clearance and oral F (bioavaiability) are means from *n* = 3. Lung *t*_½_ was determined from the PK data shown in Figure4, with *n* = 2/time point. Lung/blood split was calculated as the ratio between lung concentration 24 h after an intratracheal dose and blood *C*_max_ after the same dose. f_u_, free unbound drug in plasma; ND, not determined; logD, lipophilicity.

**Table 2:** Adenosine receptor in vitro potency

Compound	A_2a_ human CHO-K1 cells			rTNF*α* splenocytes		
	EC_50_ (nmol/L)	Intrinsic activity (%)	*n*	IC_50_ (nmol/L)	Intrinsic activity (%)	*n*
CGS21680	62.8 (16.8)	94.3 (7.8)	2	6.9 (2.8)	63.5 (8)	8
UK-432,097	5.4 (1.8)	94.8 (5.5)	3	2.6 (1.2)	60.9 (2.6)	2
1	2.4 (0.3)	102 (6.7)	7	3.2 (2.5)	53.4 (4.3)	4
2	1.4 (0.5)	97.9 (4.9)	7	0.6 (0.1)	57.4 (5.4)	6
3	0.4 (0.2)	96.2 (12.9)	3	0.2 (0.1)	58 (10.5)	4

EC_50_ and intrinsic activity values correspond to averages, standard deviations are in brackets. EC_50_, half maximal effective concentration; IC_50_, half maximal inhibitory concentration.

**Table 3:** Compound 2 selectivity and human A_2a_ potency

Compound	A_1_ human		A_2b_ human		A_3_ human		Human TNF*α* PBMC		
	EC_50_ (*μ*mol/L)	*n*	EC_50_ (*μ*mol/L)	*n*	EC_50_ (*μ*mol/L)	*n*	IC_50_ (nmol/L)	Intrinsic activity (%)	*n*
2	2.1 (1.2)	4	3.6 (2.4)	3	6.6 (3.3)	4	0.5 (0.09)	72.4 (2.5)	2

EC_50_ values correspond to averages, standard deviations are in brackets. Assay methods for A_1_, A_2b_ and A_3_ human in Data S1. PBMC, peripheral blood mononuclear cells; EC_50_, half maximal effective concentration; IC_50_, half maximal inhibitory concentration.

### In vivo PK properties of compound 2

**Figure 5:**
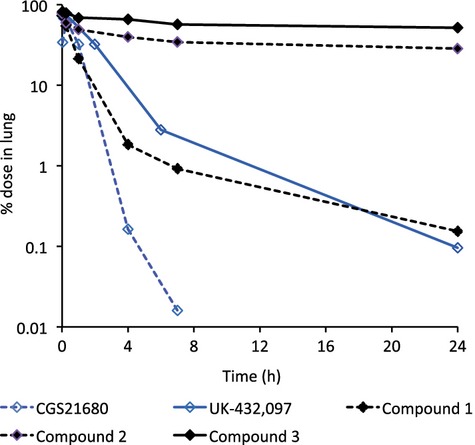
Different lung retention by inhaled A_2a_ agonists. Percentage of dose remaining in lung tissue following intratracheal administration of CGS21680, UK-432,097 and Compounds 1, 2 and 3 to rats. The dose was 300 *μ*g/kg for CGS21680, 130 *μ*g/kg for UK-432,097 and 17, 17 and 20 *μ*mol/kg for Compounds 1, 2, and 3, respectively. Data are means of *n* = 2/time point.

The PK properties of CGS21680, UK-432,097 and the three in-house compounds (1, 2 and 3) were studied in rats following i.v., oral and intratracheal administration (Table1). The concentration versus time profiles in lung and blood following i.t. dosing are depicted in Figure4, and the percentage of dose remaining in lung tissue derived from the lung concentration data are shown in Figure5. The terminal half-life in lung and lung/blood split, defined as the ratio between the total concentration in lung homogenate 24 h after dose and total blood *C*_max_ after the same dose, were calculated for each compound following i.t. dosing. CGS21680 had minimal lung retention with less than 1% of dose remaining in lung at 4 h after a single dose and a half-life in lung estimated to 1 h, and accordingly the concentration in lung at 24 h was below blood *C*_max_, that is, no lung/blood split was observed for this compound. UK-432,097 had somewhat higher lung retention with approximately 3% of dose remaining in lung at 6 h, and a lung/blood split of 2.7-fold. Compound 1 had a lung-half life of 6 h, which was dramatically increased to 53 h for its positively charged analog, compound 2, with 28% of dose remaining in lung at 24 h and a lung/blood split of 460-fold. Similar to compound 2, the positively charged compound 3 had good lung retention (lung *t*_½_ 80 h and 52% of dose remaining in lung at 24 h, lung/blood split of 1800-fold).

### Compound 2 at anti-inflammatory doses caused systemic effects

**Figure 6:**
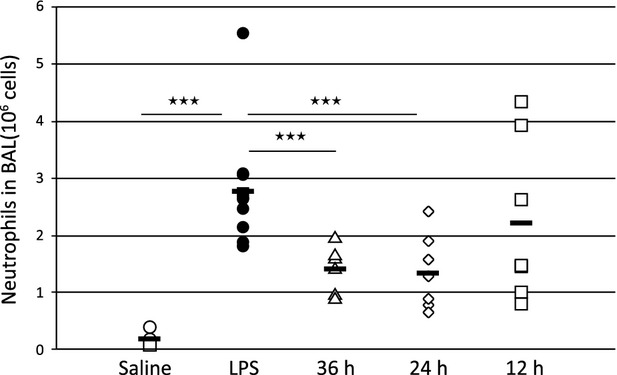
Twenty-four hours pre-treatment optimizes the anti-inflammatory effect by compound 2 given i.t. Amount of neutrophils in BAL fluid in saline-treated (open circles), LPS-challenged (closed circles) and compound 2-treated rats at 10 *μ*g/kg; 36 h (open triangles), 24 h (open diamonds) or 12 h (open squares) prior to the LPS challenge. Data is shown as individual values with group averages as a dash.

LPS induced a significant increase of neutrophils in the rat BAL fluid 24 h after LPS challenge (Fig.6). Previous dose–response studies with compound 2 had demonstrated that a single i.t. dose of 10 *μ*g/kg inhibited neutrophil recruitment in rodents to a maximum (corresponding to 40–50% inhibition). Prophylactic treatment with compound 2 at 10 *μ*g/kg showed a significant reduction of both total cells and neutrophilia in the BAL fluid with 36 and 24 h pre-treatment, whereas with 12 h pretreatment the effect was not significant (Fig.6). Therefore, a pretreatment time of 24 h was used for the upcoming studies.

**Figure 7:**
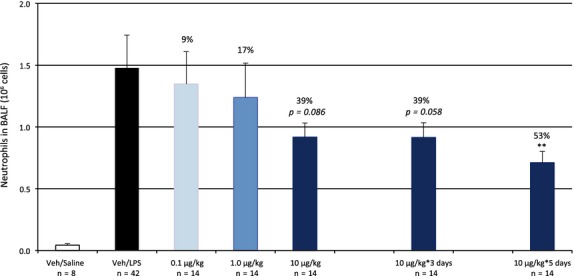
Increased and repeated doses of compound 2 increases the anti-inflammatory efficacy. Single dosing of compound 2 induced a dose-dependent decrease in the LPS-induced recruitment of neutrophilia to the lung by up to 39% (n.s.) at 10 *μ*g/kg (dark grey bar vs. LPS-challenged controls in black bar). Repeated dosing at 10 *μ*g/kg for 3–5 days increased the efficacy of the compound to a statistically significant effect vs. time-matched controls (not shown) of 53% after 5 days of dosing (*P = *0.01). Data are shown as Mean±SEM.

The present study, where three doses (0.1, 1 and 10 *μ*g/kg) were given 24 h prior to the LPS challenge, demonstrated a clear, however not statistically significant, dose–response anti-inflammatory effect by compound 2 (Fig.7, first five bars to the left), giving 9, 17 and 39% inhibition of the recovered neutrophils in the BAL fluid, respectively (*P *= 0.086 at 10 *μ*g/kg). The baseline levels of recovered cells were normal, however on the low side*,* thus giving a smaller window for the treatment effect.

Compound 2 at 10 *μ*g/kg was also given over 3 and 5 days of consecutive dosing in order to investigate a possible effect of tachyphylaxis by continuous A_2a_ receptor stimulation. There was a clear anti-inflammatory effect by compound 2 after both three and five consecutive days of dosing (Fig.7, last 2 bars), demonstrating 39% (*P = *0.058) and 53% (*P* = 0.002) inhibition of the recovered neutrophils in the BAL fluid, respectively. Consequently, the 10 *μ*g/kg i.t. dose of compound 2 was deemed the effective dose in the LPS model.

**Figure 8:**
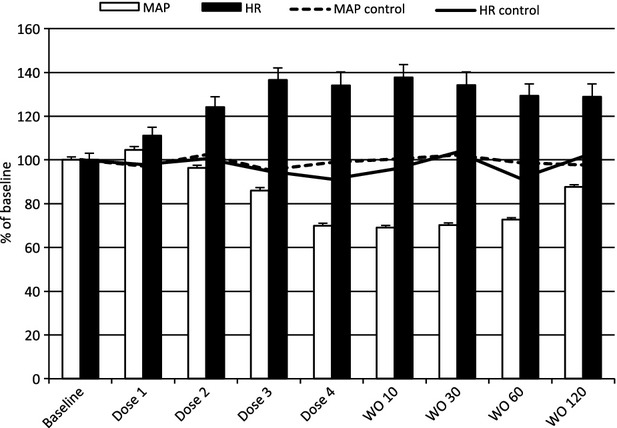
Tachycardia comes before hypotension as the plasma concentration of compound 2 increases. Blood pressure (white bars) and heart rate (black bars) in 6 conscious rats during a 4-step intravenous escalating design (Dose 1–4, equivalent to 1.9, 11, 67, and 410 *μ*g/kg per min) followed by 120 min of washout (WO 10–120) demonstrated baroreflex-compensated hypotension in response to compound 2. The dotted and solid lines represent an untreated time-match control rat in the same setting. Data are Mean±SEM for the last 5 min at each dose level.

In the swivel study, we aimed to investigate the effects on blood pressure and HR in conscious rats, and generate data suitable for PK–PD modelling by infusing compound 2 intravenously as a four-step design (Dose 1–4, equivalent to 1.9, 11, 67 and 410 *μ*g/kg per min for 15 min at each step). The main findings of the study were tachycardia and hypotension already at low plasma concentrations of compound 2, but also fatigue and a significant reduction of core body temperature at the highest dose. HR was statistically increased from 381 ± 21 to 472 ± 33 bpm at dose 2 (*P < *0.001), whereas the blood pressure effects were significantly lower from baseline at dose 3 (114 ± 9 vs. 98 ± 9 mmHg, respectively, *P = *0.006). Neither blood pressure, nor HR was returned to normal 2 h postdosing of compound 2 (100 ± 7 mmHg and 489 ± 41 bpm, *P < *0.01). Figure8 demonstrates the MAP and HR time-course for the treated rats versus one time-matched control rat.

**Figure 9:**
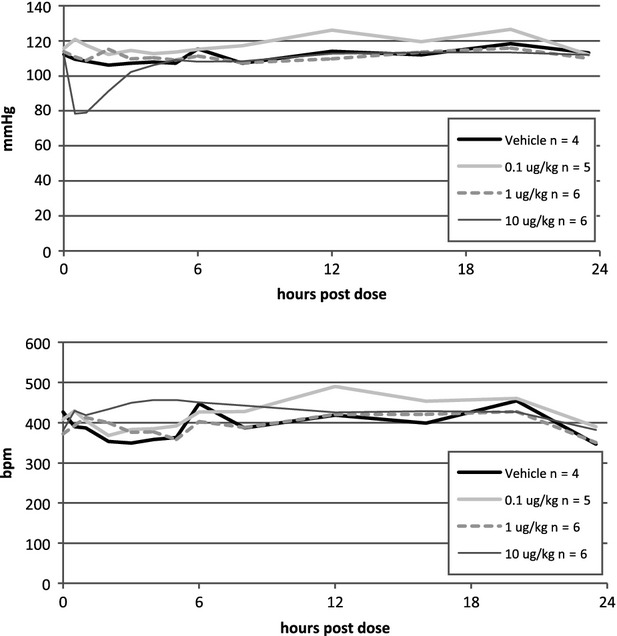
Hemodynamic responses to increasing doses of compound 2 are dose dependent. Blood pressure (top panel) and heart rate (bottom panel) responses to the first day of intratracheal (i.t.) instillation of compound 2 at 0.1 *μ*g/kg (light grey), 1.0 *μ*g/kg (dotted), 10 *μ*g/kg (dark grey) and vehicle (black) in conscious, telomerized rats demonstrated a major hypotensive response to the highest dose that was normalized within 4 h after dosing. Heart rate was significantly increased during the same time frame for 10 *μ*g/kg vs. the other groups.

In the telemetry study, we investigated the effects on blood pressure and HR following repeated once daily i.t. dosing for five consecutive days of compound 2 at 0.1, 1, and 10 *μ*g/kg. Figure9 demonstrates the blood pressure (upper panel) and HR (lower panel) effects after the first day of dosing where 10 *μ*g/kg was the only dose to induce a significant change (*P < *0.0001). The interaction between treatment group and time after dose was driven mainly by the high dose group and by the first 4 h after dosing and tells that different treatment groups show different patterns over time after dose.

**Figure 10:**
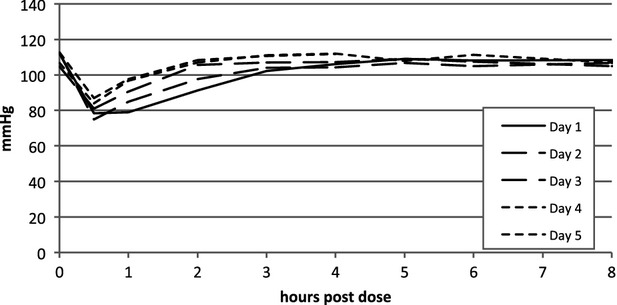
Reduced hypotensive responses after repeated dosing of compound 2. Repeated dosing of compound 2 at 10 *μ*g/kg (i.t.) in conscious, telemetrised rats demonstrated a decreased hypotensive response from day 1 to day 5 through a significant interaction between time after dose and number of doses (*P = *0.0005).

The postulated hypothesis that repeated dosing would decrease the effect on blood pressure was shown through a significant interaction between time after dose and number of doses (Fig.10, P *=* 0.0005). A significant hypotensive response was observed after the first dose of 10 *μ*g/kg, and this drop in blood pressure decreased during the following days of dosing, however it was still significant on the last day. We did not demonstrate any tachyphylaxis for the HR over the 5 days of repeated dosing (data not shown).

### PK–PD analysis of MAP and HR responses to compound 2

**Figure 11:**
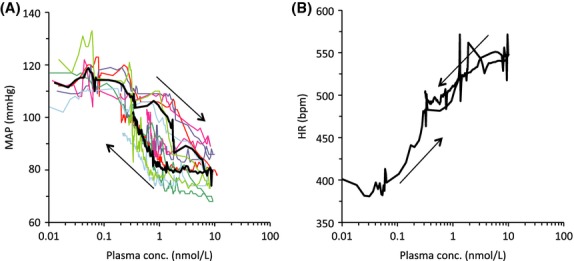
Tachycardia and hypotension are observed at increasing concentrations of compound 2 in conscious rats. The effects of four consecutive 15-min intravenous infusions of compound 2 on MAP (A) and HR (B). Plasma concentrations were simulated based on modeling of observed PK data. Individual data are shown with thin coloured lines (A) and mean data (*n* = 6) are shown with a thick black line (both A and B). Arrows denote time order of measurements.

The relationship between compound 2 plasma concentrations and MAP and HR, respectively, were modeled using data generated in the swivel study. Three of the seven rats demonstrated a HR increase during the first infusion step that was judged as stress or activity related and not associated with the drug infusion, and the data points during the first infusion were discarded for the PK–PD analysis. Hysteresis was observed when individual concentration–MAP response data were plotted in time order (Fig.11). To account for the concentration-effect-time delay an effect-compartment model linked to a decrease in MAP according to a sigmoid *E*_max_ model was used to model the data for each individual rat, yielding mean ± SD parameter estimates of 0.058 ± 0.039/min for *k*_*E0*_ (first-order rate constant), 114 ± 6 nmol/L for E0,MAP (the baseline MAP), 37 ± 11 nmol/L for *Emax,MAP* (the maximum compound 2-induced decrease in MAP), 0.52 ± 0.16 nmol/L for *EC50,MAP* (the concentration that induces half the maximum MAP decrease) and 1.8 ± 1.1 for *n* (the Hill factor). HR data were not possible to model on an individual basis with satisfactory fit, but the mean concentration-mean HR response data could be described by a simple *E*_max_ model, yielding estimates of 386 nmol/L for E0,HR (the baseline HR), 158 nmol/L for Emax,HR (the maximum compound 2-induced increase in HR) and 0.18 nmol/L for EC50,HR.

Based on the PK–PD relationships, the total plasma concentration of compound 2 that causes 10% decrease from baseline MAP was calculated to be 0.3 ± 0.2 nmol/L, whereas the concentration that produces 10% increase from baseline HR was calculated to be 0.04 nmol/L.

### Rat cardiomyopathy caused by compound 2

**Figure 12:**
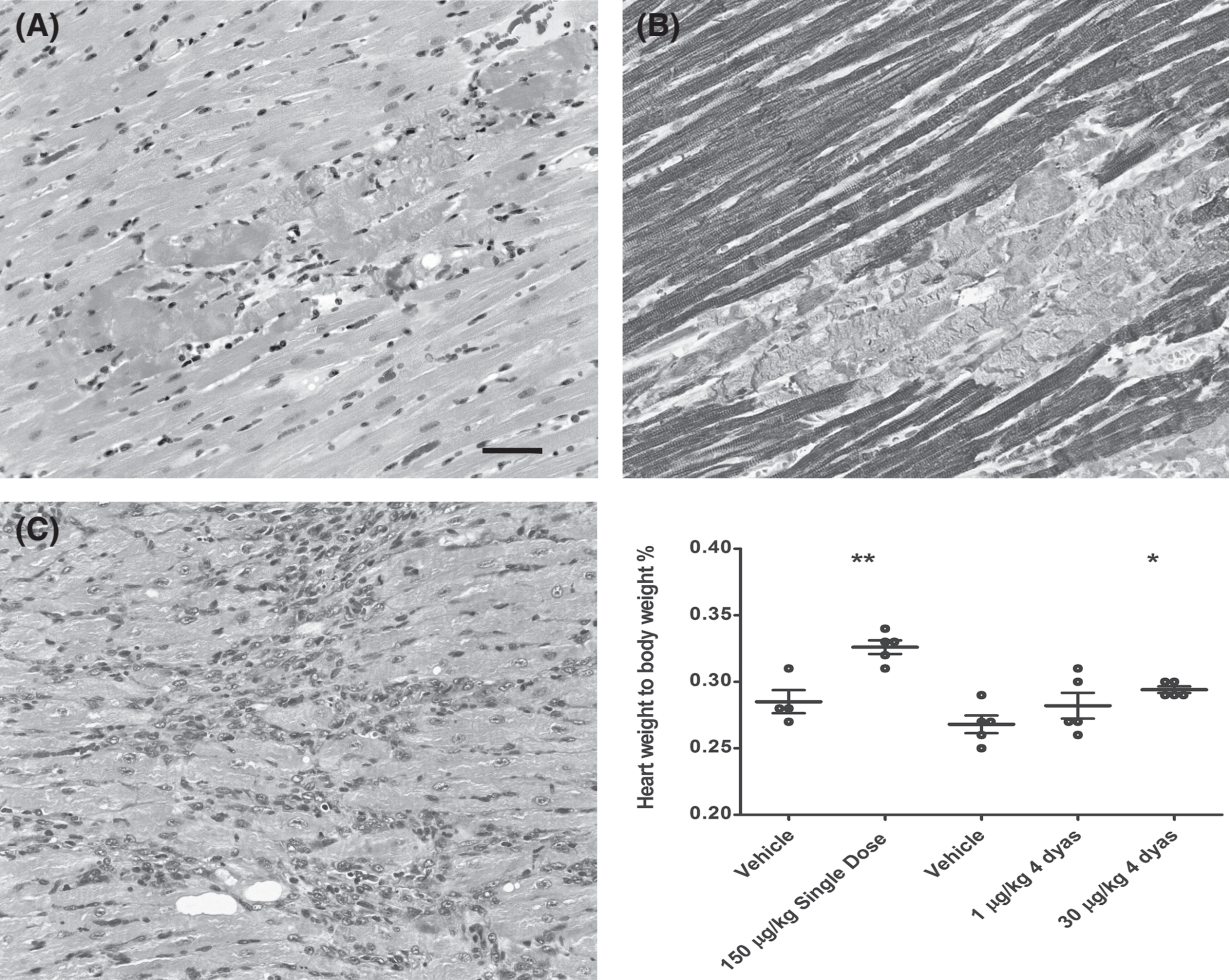
The heart histopathology and heart weight are altered by compound 2. Rats received a single dose of compound 2 (150 *μ*g/kg) had multifocal myocardial necrotic areas in the heart (A). In the acute damaged areas cardiomyocytes had blurred cellular boundary and eosinophilic cytoplasm. The necrotic areas had negative staining of Troponin T (B). Rats received compound 2 (30 *μ*g/kg) for 4 days had multifocal dense granulation tissue with early scar formation (C). The heart weight was increased in the animals with myocardial lesions. SD, single dose; 4D, dose for 4 days. The bar represents 200 *μ*m.

One day after receiving the i.t. single dose of compound 2 (150 *μ*g/kg), four of five animals in the group showed multifocal myocardial necrosis (Fig.12A). In these necrotic areas, cardiomyocytes had eosinophilic or pale cytoplasm, patchy loss of cross striations, wavy and blurred cellular boundaries, frequently surrounded by a few inflammatory cells. These areas were randomly distributed in the heart, although more commonly seen in the endocardial regions. In addition, perivascular and/or ventricular edema were also observed in these animals. Immunohistochemistry stain confirmed that Troponin T was negative in the cardiomyocytes of the necrotic regions (Fig.12B).

In the repeated dose study (30 *μ*g/kg for 4 days), three out of five animals that received compound 2 had multifocal “scar” tissue appearing as dense granulation with different cell types (e.g., fibroblasts, macrophages and capillaries) and minimal collagen deposition (Fig.2C). Corresponding to these histopathologic changes, the heart weight, presented as relative heart weight to the body weight, was found to be slightly increased in animals that received either a single dose of 150 *μ*g/kg or repeated doses of 30 *μ*g/kg (Fig.2). No changes were observed in animals that received 1 *μ*g/kg for 4 days.

## Discussion and Conclusions

The present study demonstrates the challenges in achieving a therapeutic window between the beneficial anti-inflammatory responses in the lung and the undesired cardiovascular effects even after local lung administration.

Synthetic A_2a_ agonists (e.g., CGS21680 and UK-432,097) and antagonists (e.g., ZM-241,385) have been developed and characterized in recent years (Fig.1). CGS21680 administered intratracheally to allergen-challenged Brown Norway rats inhibited lung inflammation, however the same doses caused hypotension and tachycardia (Fozard et al. 2002). All observed effects by CGS21680 were prevented by pretreatment with the selective A_2a_ antagonist ZM-241,385. The prominent role for the A_2a_ receptor in the CGS21680-induced hypotension and reactive tachycardia has been extensively studied and is now established (Webb et al. 1990, 1991; Ledent et al. 1997).

UK-432,097 is an inhaled potent A_2a_ agonist with selectivity over A_2b_ and A_1_, but it bears an agonistic effect on the A_3_ receptor. This compound showed low oral bioavailability, low solubility, rapid clearance, and high plasma protein binding (Mantell et al. 2010). Due to the lack of effect in an inflammatory model with a neutrophil-dependent end point, a guinea pig model with capsaicin-induced bronchoconstriction was used for preclinical efficacy testing (Mantell et al. 2008; Trevethick et al. 2008). UK-432,097 had no safety limitation over the dose range tested in the Phase I clinical study but Phase II study failed to show efficacy in patients with COPD (Mantell et al. 2009, 2010).

As A_2a_ governs both anti-inflammatory (lung/airways) and cardiovascular (systemic circulation) mechanisms, we hypothesized that it should be possible to achieve an acceptable therapeutic window for a compound demonstrating good lung retention, such that a high ratio of lung concentration to blood concentration after dosing to the lung was achieved. Our strategy focused on potent and selective A_2a_ agonist with increased lung retention and drug-like properties differentiated from UK-432,097.

Our chemistry aimed to maintain the adenine skeleton and also to keep the ribose moiety, as *N*-ethyl-*β*-D-ribofuranosyl-uronamide, which is known to be one of the most potent ribose modifications (Müller and Jacobson 2011), whilst optimizing the C2 and N6 positions (Fig.2). Several substituents were screened; 1-ethylpropyl and ethyl urea pyridine in C2 and N6 position, respectively (compound 1) were found to be optimal for potency and selectivity with good logD and solubility. The ethyl urea pyridine provided a handle to further elaborate on physical chemical properties of the compounds to optimize lung retention (Tables3, Fig.3).

**Figure 13:**
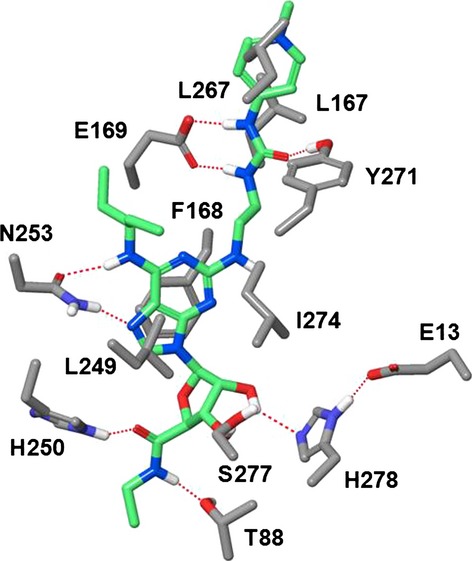
Binding mode of compound 2 in the X-ray structure of human A_2a_ in the agonistic conformation. Residues critical for potency and selectivity are indicated. The urea fragment of the C2-side chain is H-bonded to E-169 and Y-271. The terminal positively charged guanidinium moiety is exposed to the extracellular environment and squeezed between residues L167 and L267. H-bonds are shown by red dotted lines. Carbon atoms of compound 2 are shown in light green. Nonpolar hydrogens are not shown for clarity.

There is scarce public information on molecular drivers to achieve optimal lung retention. It has previously been shown that basic and dibasic compounds may in some cases provide good retention in the lung (Cooper et al. 2012), albeit this does not apply in all cases and is difficult to predict. Compound 2 was designed from compound 1 by methylating the pyridine giving a highly soluble compound with extended rat lung *t*_½_, increased potency, maintained selectivity profile, very low logD and low plasma protein binding (Table1). Figure13 illustrates the binding mode of compound 2 docked into the X-ray A_2a_ crystallographic structure in the agonistic conformation (Lebon et al. 2011; Deflorian et al. 2012). The positively charged pyridinium ring with the directly coupled urea creates a cationic conjugated system that forms a strong ionic H-bond to E-169 and an additional H-bond to Y-271. Therefore, the C2 side chain in compound 2 underlines dramatic improvement of rat lung retention together with gain of new interactions between A_2a_ receptor and compound 2.

Compound 3, containing a pyridium ion in the C2 chain and the diphenyl substituent on the N6 position, was further synthesized (Fig.3) for comparison with UK-432,097. Both compounds 2 and 3 (Fig.3, Tables1 and 2) showed good lung retention with high separation between lung and blood drug concentration. Compound 2 was selected for further in vivo profiling based on overall properties profile, as example, A_2a_ potency was more than 1000-folds over A_1_, A_2b,_ and A_3_.

Molecular modeling suggested that the binding mode of compound 2 in the adenosine A_2a_ receptor (Fig.3) is very similar to that of UK-432,097. The H-bonding pattern and hydrophobic interactions of the minimum-energy structure are identical to those observed by the X-ray crystallographic studies (Xu et al. 2011). The terminal positively charged guanidinium moiety of compound 2 is exposed to the extracellular environment and squeezed between side chains of L167 and L267, the specific residues of the A_2a_ receptor.

A very high percentage of the lung deposited dose of compound 2 remains in the lung throughout the period studied after dose (Fig.5). Compound 2 is a permanently positively charged high-molecular-weight substance and it could be surmised that poor permeability, through the epithelial lining of the airways into the lung tissue and subsequently into the systemic circulation, could give rise to the high observed lung retention. Low permeability and slow passage into lung tissue could be supported by the onset of inhibition of LPS stimulated recruitment of neutrophils after i.t. administration of compound 2 in the in vivo experiments, where the optimal pretreatment time was between 24 and 36 h for compound 2 (Fig.6). This was not observed for the rapidly absorbed compound CGS21680 (Table1, Figs4 and 5) where the maximal efficacy was seen already after 30 min pretreatment (data not shown).

However, other permanently charged (quaternary nitrogen) compounds, such as tiotropium, are well absorbed following inhalation (approximately only 10% of the lung deposited dose remains in the lung 2–4 h after dosing) (Cooper et al. 2012), supporting that membrane permeability is not necessarily limiting the absorption from the airways for permanently charged molecules.

Based on the long lung retention and high lung concentrations of compound 2 in relation to its in vitro potency, the efficacious dose in the LPS-induced lung inflammatory model was expected to be lower than observed. Although the reason is not understood, our data indicates that the properties of compound 2 makes a majority of the compound given by i.t. dosing distribute into lung tissue compartments that appear to be inaccessible to the A_2a_ receptor on the target cells in the lung. It cannot be excluded that the compound measured in lung tissue homogenate may include amounts remaining in the airways that have not been absorbed into the lung tissue.

The plasma concentration of compound 2 that induced a 10% decrease of MAP was estimated to be 0.3 nmol/L, which is 10-fold lower than the plasma concentration of 2–4 nmol/L observed 10 min after i.t. dosing of compound 2 at the effective dose level of 10 *μ*g/kg in the LPS model (PK data, not shown).

The measured systemic plasma exposures that caused hypotensive effects were in the same range as the in vitro potency. Several papers have been published on different A_2a_ agonists that demonstrate the same relationship between in vitro potency and vasodilatation (Webb et al. 1990, 1991; Mathôt et al. 1995; Dhalla et al. 2006). To explore if the safety window would be increased with repeated dosing, we studied the hemodynamic responses over five consecutive days after once daily intratracheal instillations of compound 2 in conscious, unrestrained rats. We found that the hypotensive effect actually decreased significantly over the 5 days of 10 *μ*g/kg dosing, however, there was still a significant hypotension observed on the last day versus vehicle. These results do not support the development of tolerance of the blood pressure effects reported by Monopoli et al. (1998). Furthermore, in the swivel rat model we observed effects on HR at plasma concentrations that were below those that caused effects on MAP. The baroreflex mechanism was not studied in the present study, but it is likely that the observed tachycardia is mainly driven by this mechanism (Webb et al. 1990, 1991; Mathôt et al. 1995), although despite of the extensive selectivity screening it cannot be out-ruled that compound 2 has other activities that may lead to an increase in HR (Dhalla et al. 2006).

Our toxicological study demonstrated that intratracheal administration of compound 2 with a single dose (150 *μ*g/kg) caused multifocal myocardial necrosis in rats. Similar types of myocardial degeneration and necrosis have been observed in toxicological studies with oral A_2a_ agonists, such as CI-947 in a monkey study (Albassam et al. 1998) and the A_2a_/A1 agonist DPEA in a dog study (Macallum et al. 1991). These oral A_2a_ agonists often showed coronary arterial lesions which were missing in our toxicological study after local airway administration. In the repeated dose group (30 *μ*g/kg for 4 days), there was multifocal dense scar tissue in the heart but no acute myocardial necrosis, suggesting that there was a decreased incidence of acute ischemic lesions by the later doses of compound 2 in the animals. This different pattern of cardiomyopathy after single and repeated doses taken together with the absence of coronary arterial lesions indicate a possible link between the tachyphylaxis of hypotensive reponses to the myocardiopathy of this A_2a_ agonist.

In conclusion, a new potent and selective A_2a_ agonist was discovered and this compound demonstrated significantly increased lung retention, high lung to systemic exposure and differentiated physico-chemical properties from the known clinical A_2a_ candidate UK-432,097. The above novel features of compound 2 did not provide a sufficient therapeutic window to separate the anti-inflammatory effect from cardiovascular effects in preclinical models.

## Abbreviations

COPD: chronic obstructive pulmonary disease
BAL: broncheoalveolar lavage
EC_50_: half maximal effective concentration
F: bioavailability
f_u_: fraction unbound
HPLC: high-performance liquid chromatography
HR: heart rate
i.t.: intratracheal
i.v.: intravenous
IC_50_: half maximal inhibitory concentration
logD: lipophilicity
LPS: Lipopolysaccharide from *Pseudomonas Aeruginosa* serotype 10
MAP: mean arterial blood pressure
p.o.: per oral
PBMC: peripheral blood mononuclear cells
PD: pharmacodynamic
PK: pharmacokinetic

## Supporting Information

**Data S1**. Supplementary Materials for The Discovery of a Selective and Potent A2a Agonist with Extended Lung Retention.
