# Supplementary material for: The discovery of a selective and potent A2a agonist with extended lung retention
Source: Pharmacol Res Perspect. 2015 May 4;3(3):e00134. doi: 10.1002/prp2.134 (PMC4492750; doi:10.1002/prp2.134)
Supplement: Supplementary file 1 [file prp20003-e00134-sd1.docx]

**Supplementary Materials for**

**The Discovery of a Selective and Potent A_2a_ Agonist with Extended Lung Retention**

**ABM Åstrand^1*^, E Lamm-Bergström^1^, H Zang^2^, L Börjesson^1^, T Söderdahl^2^, C Wingren^1^, AH Jansson^1^, A Smailagic^1^, C Johansson^2^, H Bladh^3^, I Shamovsky^1^, A Tunek^1^, T Drmota^1^**

AstraZeneca R&D Mölndal, ^1^RIA iMed and ^2^Drug Safety & Metabolism, SE-431 59 Mölndal, Sweden, ^3^AstraZeneca R&D Lund, SE-221 87 Lund, Sweden.

**Running title: Potential for A2a agonism in respiratory inflammation**

^*^To whom correspondence should be addressed: Dr Annika Åstrand, AstraZeneca R&D Mölndal, SE-43183 Mölndal, Sweden. Tel.;+46317761620; Fax; +46317763852; Email: [annika.astrand@astrazeneca.com](mailto:annika.astrand@astrazeneca.com)

**This PDF file includes:**

**Methods (details)**

**METHODS**

***In vitro assays***

*Human A_2a_ and A_2b_ receptor pharmacology*

Chinese hamster ovary CHO-K1 cells stably expressing human A_2a_ and A_2b_ receptors were stored as 1ml aliquots at -150^o^C in freezing medium, thawed immediately prior to assay. To determine substances agonistic potency and efficacy, the generation of cAMP was measured by an Alpha screen assay (cAMP Alphascreen assay kit, Perkin Elmer 6760625M; Greiner Lumitrac 200 white 384-well assay plate Greiner #781075; Envision 2102 Multiple reader - Perkin Elmer) according to the manufacturer experimental protocol in assay buffer (1xHBSS, 5mM Hepes pH 7.4, 40µM Rolipram (Sigma R6520), 0.1% (w/v) BSA). Cells (10000 cells/well) were incubated with test compounds prepared as serial dilutions. Potency (EC_50_) and efficacy (% of maximal response) were calculated from data fitted to a 4-parameter logistic function. The signal of 10µM NECA represented the maximal cAMP response (*i.e.* 100% of efficacy) produced by these cells.

*Human A_1_ receptor pharmacology*

A membrane preparation expressing the Adenosine A_1_ Receptor (Perkin Elmer ES-010-M400UA) was used to determine the substances agonistic potency and efficacy. The A_1_ receptor G_i_ coupling was measured by a scintillation proximity (SPA) GTPγ ^35^[S] assay. Membranes (12.5μg/ml final concentration) were incubated with substances in serial dilutions (50mM Hepes, pH 7.4, 1mM MgCl_2_, 100mM NaCl, 0.01% BSA, 50μg/ml saponin and 2.5μM GDP) including 1mg/ml Scintillation Beads (Wheat Germ Agglutinin PVT SPA, Perkin Elmer - Rpnq0001), 0.3nM of GTPγ ^35^[S] (Perkin Elmer - NEG030H) and counts were read after 16h incubation at 4^o^C. The signal corresponding to adenosine at 100μM was used to define 100% efficacy.

*Human A_3_ receptor pharmacology*

A_3_ receptor G_i_ coupling was measured by GTPγ ^35^[S] filter assay using membrane from Perkin Elmer (ES-012-M400UA). Membranes (7.5μg/ml final concentration) were incubated with substances in serial dilutions (50mM Hepes, pH 7.4, 5mM MgCl2, 100mM NaCl, 0.01% BSA, 100μM DTT and 25μM GDP) including 1nM of GTPγ ^35^[S] (Perkin Elmer - NEG030H) for 2h in room temperature in the assay plate (Corning – 3365). After incubation, 200μl was transferred to a filter plate (UniFilter-96, GF/C, Perkin Elmer – 6005174) and washed 15 times with ice cold wash buffer (50mM Tris, pH 7.4, 5mM MgCl_2_ and 50mM NaCl). After adding 50µl scintillant/well, sealed plates were counted. The signal corresponding to the maximal response of NECA at 100μM was set to 100% efficacy.

*LPS-induced TNFα production in primary human Peripheral Blood Mononuclear Cells (hTNFα PBMC) and rat mononuclears (rTNFα splenocytes)*

Procedures and assays were run in R10 buffer (RPMI 1640 Medium supplemented with GlutaMAX™ and HEPES (GIBCO-BRL), 10% fetal calf serum, penicillin and streptomycin (each 5000IU/ml)). Human PBMC were purified from heparinised blood by Ficoll separation (Ficoll, Amersham Pharmacia Biotech AB, Cat: 17-1440-03, Lot: 312170) and rat mononuclear cells were collected after rat spleen perfusion. Both cell preparations were followed by hypotonic lysis of residual erythrocytes. Serial dilutions of compounds were pre-incubated with 200 000 cells/well (96-well plate) for 45min in 37^o^C (190µl). Cells were stimulated by 10µl LPS (final concentration 0.5ng/ml human and 150ng/ml rat; SIGMA Cat: L-4516, Lot: 104K4036) for 18h at 37^o^C. TNFα release was measured by ELISA (^Tm^R&D Systems plates) according to the manufacturer’s instructions in cell free supernatant.

*Plasma protein binding*

The plasma protein binding was determined in plasma from male Wistar rats at a compound concentration of 10μM by equilibrium dialysis against a phosphate buffer at pH 7.4 using a semi-permeable membrane with a molecular cut-off of 6-8KDa (Spectra/Por, Spectrum Laboratories Inc, US). The compounds were dialyzed for 18h at 37±1°C in an air incubator on an orbital shaker. The samples were analysed using LC-MS/MS.

*Lipophilicity*

The octanol-water partition coefficient (LogD) was measured using the shake flask method. 600µL of 1-octanol was added to a vial with 6µL of 10mM DMSO-solution of the test compound. 600µL of 10mM sodium phosphate buffer adjusted to pH 7.4 was added and equilibrium was performed by vigorous shaking for ten minutes with an Eppendorf mixer. The phases were separated by centrifugation. The samples were analysed by HPLC with UV-detection at 254nm.

*Substance solubility*

Solubility was measured in phosphate buffer at pH 7.4 using dry compounds in solid state.

***In vivo assays***

*In vivo Pharmacokinetics studies*

Rat PK was determined after an *i.v.* dose of 0.1μmol/kg (approximately 50μg/kg, 1ml/kg, TEG:H_2_O 50:50) given via a catheter in *vena jugularis*. The PK after oral administration was performed at a dose of 6μmol/kg (approximately 3mg/kg, 4ml/kg, 0.5% HPMC) given by gavage. Intratracheal PK was determined at 0.03 to 0.6μmol/kg (17 to 300μg/kg, 1ml/kg, in a vehicle containing sodium chloride (8.5mg/ml), EDTA (0.1mg/ml), citric acid (0.15mg/ml), sodium citrate (0.5mg/ml) and polysorbate 80 (0.2mg/mL) in purified water). The *i.t.* dose was given to anaesthetized (isofluran) rats, positioned at approximately 30° angle (head up) and the compound was administered in the trachea using a modified metal cannula. Rats were put back in their cages while regaining consciousness. Serial blood sampling of 100 to 200µl was performed after *i.v.* and *p.o.* dosing at pre-defined timepoints. Blood was mixed 1:4 with purified water containing heparin (10 to 30U/ml) in order to haemolyse the blood cells, and then placed in a 96-well plate and stored at –70°C until analysed. The dilution factor was accounted for in the data analysis. After *i.t.* dosing, animals were euthanized with isoflurane or pentobarbital at defined timepoints after dosing, blood samples taken from the *vena jugularis* or *arteria carotis*, handled and stored as described above. The rats were bled to drain as much blood as possible from the lungs. The lungs including the trachea were carefully dissected out with all non-relevant tissue removed, placed in pre-weighed vials and weighed. The lung vials were stored at –70°C until analyzed.

*In vivo Pharmacodynamic studies*

Animals in all efficacy studies were dosed intratracheally as described above in a buffered saline vehicle (pH 5) containing sodium chloride (8.5mg/ml), citric acid (0.28mg/ml) and sodium citrate (0.5mg/ml). The compound was dissolved in saline when given intravenously (0.9% NaCl) to the swivelled rats.

*LPS challenge*

The LPS intratracheal instillation was performed 24 hours after a single dose or the last dose of compound 2 as described in the supplementary material. Each rat was given LPS from the *Pseudomona Aeruginosa* bacteria (1µg/rat, 5µg/ml saline, Sigma-Aldrich, Sweden).

*Termination and broncheoalveolar lavage (BAL) procedure*

24 hours after the LPS challenge, rats were anaesthetized and exsanguinated via a cardiac incision. A tube was inserted and ligated to the trachea. The tube was connected to a syringe with 4ml PBS (without Ca^2+^/Mg^2+^) and a lavage of the whole lung was performed manually. The BAL fluid was collected individually in polyethylene tubes and kept on ice until further processed.

*Preparation of BAL fluid for cell count and mediator analysis*

The BAL fluid was centrifuged and the supernatant separated from the cell pellet that was resuspended in 1.0ml of PBS. The total number of cells and cell differentia was counted in an automated cell counter and haematology analyzer (SYSMEX XT-1800iV, software for the rat BALF).

*Rats in the Swivel (tether) system*

A 4-step intravenous infusion protocol was performed during continuous recording of the arterial blood pressure signal and intermittent blood sampling in seven conscious rats the day after surgical implantation of an arterial line in the abdominal aorta via the caudal artery and a venous line in the jugular vein. After a baseline recording of 60 minutes, compound 2 was given in 15-minute constant infusion steps of 1.9, 11, 67 and 410μg/kg/min (0.034-0.2ml/kg/min) followed by a washout period of 120 minutes. One rat was monitored under the same conditions as a time-matched control. Blood samples for analysis of drug exposure were taken at the end of each dose step and intermittently during the washout.

*Rats in the Telemetry system*

Seven days before initiation of the dosing regimen, 24 rats were anaesthetized (isoflurane) and a small telemetry unit (PA-C10, Data Sciences International, USA) was placed subcutaneously on the left side of the throat with the catheter going into the left carotid artery. A baseline recording of blood pressure and heart rate was initiated at least 48 hours prior to the first dose of compound 2. The compound was given by intratracheal instillation as described above, for 5 consecutive days, and blood pressure and heart rate were continuously recorded, apart from the brief period during compound administration.

*Rat toxicology study*

Rats were intratracheally administrated with compound 2 at 150µg/kg and terminated 24 hours after dosing. Rats in the repeated dose groups received compound 2 at dose level of 1 or 30µg/kg for 4 consecutive days and were terminated 24 hours after the last dose.

***Sample processing and analysis***

*Tissue sampling and tissue processing*

The animals were sacrificed by exsanguinations from the common carotid artery under isoflurane anesthesia. The external features were inspected and body weights recorded. The lung together with lower part of the trachea was excised from the thoracic cavity, weighed, and inflated with Formalin via the trachea. Other core organs (heart, kidneys, liver and spleen) were also macroscopically examined, weighed and fixed in buffered Formalin. The collected organs were trimmed and processed to paraffin blocks, sectioned (4µm) and stained with Hematoxylin Eosin (HE) for microscopic examination. Additional heart sections from each animal were collected for immunohistochemistry of Troponin T (see below) and for Masson's Trichrome stain.

*Immunohistochemistry (IHC)*

The IHC was performed by using Ventanan Discovery XT automated slide processing system, according to the manufacturer’s instructions. In brief, after the antigen retrieval process (Std CC1), the Troponin T antibody (Thermo Scientific, MS-295-P1 diluted 1:50 in Antibody Diluent) was applied on the sections for 60 minutes, followed by incubation with rabbit monoclonal anti-mouse (Epitomics catalog no 3021-1) for 60 minutes and a polymer OmniMap-anti-rabbit HRP Multimer for 16 minutes. The reaction was visualized by using ChromoMap DAB kit. The slides were dehydrated in rising concentration of ethanol, cleared in xylene in an Automatic slide strainer (Sakura, US) and mounted manually with Mountex (00842, Histolab Products AB, Sweden).

*Microscopic examination and image capture/justification*

Specimens were examined using a microscope (AxiaoPlan 2 Imaging, Zeiss) and the images were taken by using a digital camera (DP 200) with the Picsara program (version 9.4, Euromed Networks, Sweden) and synched in Photoshop software (Adobe).

*Determination of drug concentration in lung, plasma and blood*

Lung tissue was frozen in liquid nitrogen and homogenised using a CryoPrep pneumatic hammer. The homogenisation of the tissue and the extraction of the compound were done in 4ml Ringer solution with an Adaptive Focused Acoustic Instrument. 50µl of the supernatant was transferred to a 96-well plate. All lung, blood and plasma samples were analysed by LC-MS/MS using a selective and sensitive method. Peak areas were used in comparison to those produced by the standard curve to determine parent compound concentrations. Homogenate concentrations were adjusted to account for the sample dilutions to give tissue concentrations.
